# Eplerenone ethanol solvate

**DOI:** 10.1107/S1600536808009240

**Published:** 2008-04-10

**Authors:** Qian Yang, Wei-Dong Ye, Jian-Yong Yuan, Jing-Jing Nie, Duan-Jun Xu

**Affiliations:** a Department of Chemistry, Zhejiang University, Hangzhou 310027, People’s Republic of China; b Xinchang Pharmaceutical Factory, Xinchang 312500, People’s Republic of China

## Abstract

Eplerenone [systematic name: 7α-(methoxy­carbon­yl)-3-oxo-9α,11-ep­oxy-17α-pregn-4-ene-21,17-carbolactone], an aldo­sterone receptor antagonist, crystallizes from ethanol as a monosolvate, C_24_H_30_O_6_·C_2_H_6_O. The eplerenone mol­ecule has two five-membered rings, three six-membered rings and one three-membered ring. Both five-membered rings display envelope conformations, while the three six-membered rings assume envelope (cyclohexene), half-chair (cyclohexane sharing one edge with epoxy) and chair (other cyclohexane) conformations. The solvent mol­ecule is disordered equally over two sites. It is linked to the eplerenone mol­ecule by hydrogen bonds.

## Related literature

For background literature, see: Grob *et al.* (1985 ▶). For related structures, see: Grob *et al.* (1997 ▶); Yang *et al.* (2007 ▶); Xu *et al.* (2007 ▶). For ring analysis, see: Spek (2003 ▶).
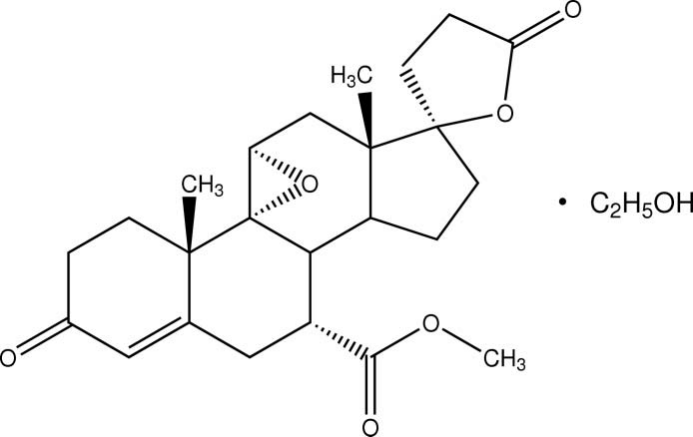


## Experimental

### 

#### Crystal data

C_24_H_30_O_6_·C_2_H_6_O
*M* *_r_* = 460.55
Orthorhombic, 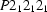

*a* = 8.3236 (5) Å
*b* = 12.8306 (9) Å
*c* = 23.3173 (13) Å
*V* = 2490.2 (3) Å^3^
*Z* = 4
Mo *K*α radiation
μ = 0.09 mm^−1^
*T* = 295 (2) K
0.20 × 0.16 × 0.14 mm

#### Data collection

Rigaku R-AXIS RAPID IP diffractometer
Absorption correction: none
19548 measured reflections
2559 independent reflections
1955 reflections with *I* > 2σ(*I*)
*R*_int_ = 0.053

#### Refinement

*R*[*F* ^2^ > 2σ(*F* ^2^)] = 0.056
*wR*(*F* ^2^) = 0.151
*S* = 1.05
2559 reflections
293 parameters
6 restraints
H-atom parameters constrained
Δρ_max_ = 0.30 e Å^−3^
Δρ_min_ = −0.18 e Å^−3^

### 

Data collection: *PROCESS-AUTO* (Rigaku, 1998 ▶); cell refinement: *PROCESS-AUTO*; data reduction: *CrystalStructure* (Rigaku/MSC, 2002 ▶); program(s) used to solve structure: *SIR92* (Altomare *et al.*, 1993 ▶); program(s) used to refine structure: *SHELXL97* (Sheldrick, 2008 ▶); molecular graphics: *ORTEP-3 for Windows* (Farrugia, 1997 ▶); software used to prepare material for publication: *WinGX* (Farrugia, 1999 ▶).

## Supplementary Material

Crystal structure: contains datablocks I, global. DOI: 10.1107/S1600536808009240/ng2439sup1.cif

Structure factors: contains datablocks I. DOI: 10.1107/S1600536808009240/ng2439Isup2.hkl

Additional supplementary materials:  crystallographic information; 3D view; checkCIF report

## Figures and Tables

**Table 1:** Hydrogen-bond geometry (Å, °)

*D*—H⋯*A*	*D*—H	H⋯*A*	*D*⋯*A*	*D*—H⋯*A*
O8—H8*A*⋯O1^i^	0.96	2.19	3.15 (4)	178
O9—H9*A*⋯O1^i^	0.98	2.34	3.32 (4)	177

Symmetry code: (i) 
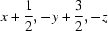
.

**Fig. 1.**
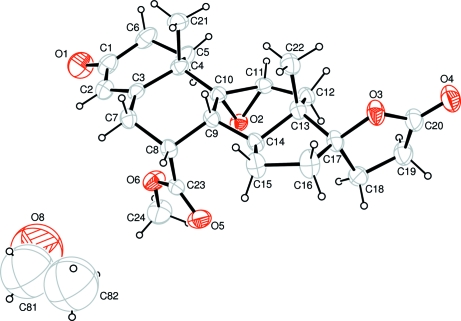
The molecular structure of the title compound with 30% probability displacement (arbitrary spheres for H atoms). One of disordered solvent components has been omitted for clarity.

## supplementary crystallographic information

### Comment

The eplerenone is known as an aldosterone receptor antagonist and can be administered in a therapeutically effective amount where use of an aldosterone receptor antagonist (Grob *et al.*, 1985). The crystal structure of the eplerenone ethanol solvate is reported here.

The crystal of the title compound consists of eplerenone molecules and lattice ethanol molecules (Fig. 1). The molecule of eplerenone contains three six-membered rings, two five-membered rings and one three-membered ring. A ring analysis (Spek, 2003) indicates that three six-membered rings assume different conformations: chair, half-chair and envelope; both five-membered rings display the similar envelope configuration. This agrees with those found in the structure of eplerenone THF solvate (Yang *et al.* 2007) and in the structure of eplerenone dioxane solvate (Xu *et al.*, 2007). The C2—C3 bond distance of 1.343 (6) Å indicates the typical C?C double bond. The C23-ester group forms an intra-molecular C—H···O hydrogen bond with the adjacent C14-methine group (Table 1). This structural feature is also found in the crystal structure of eplerenone dichloromethane solvate (Grob *et al.*, 1997).

In the crystal structure, lattice solvent molecules are disorderly located in the cavities formed by eplerenone molecules and link with eplerenone molecules *via* O—H···O and C—H···O hydrogen bonding (Table 1).

### Experimental

A microcrystalline powder sample of eplerenone was prepared in the manner reported by Grob *et al.* (1997). Single crystals of the title compound were obtained from an ethanol solution of eplerenone.

### Refinement

The lattice ethanol molecule is disordered in the crystal structure; a two-site model with each 0.5 site occupancies was adopted in the refinement. The C—C and C—O distances for the disordered solvent molecule were constrained to 1.50±0.01 and 1.40±0.01 Å, respectively; atomic displacement parameters for non-H atoms of the disordered solvent molecule were constrained to be the same. Hydroxyl H atoms were placed in chemical sensible positions and refined in riding mode with *U*_iso_(H) = 1.5*U*_eq_(O). Other H atoms were placed in calculated positions with C—H = 0.93 to 0.98 Å, and refined in riding mode with *U*_iso_(H) = 1.5*U*_eq_(C) for methyl or 1.2*U*_eq_(C) for others. In the absence of significant anomalous scattering effects, Friedel pairs were merged; the absolute configuration was not determined.

### Crystal data

**Table:**

C_24_H_30_O_6_·C_2_H_6_O	*F*_000_ = 992
*M**_r_* = 460.55	*D*_x_ = 1.228 Mg m^−^^3^
Orthorhombic, *P*2_1_2_1_2_1_	Mo *K*α radiation λ = 0.71073 Å
Hall symbol: P 2ac 2ab	Cell parameters from 4788 reflections
*a* = 8.3236 (5) Å	θ = 3.2–25.2º
*b* = 12.8306 (9) Å	µ = 0.09 mm^−^^1^
*c* = 23.3173 (13) Å	*T* = 295 (2) K
*V* = 2490.2 (3) Å^3^	Prism, colorless
*Z* = 4	0.20 × 0.16 × 0.14 mm

### Data collection

**Table:**

Rigaku R-AXIS RAPID IP diffractometer	1955 reflections with *I* > 2σ(*I*)
Radiation source: fine-focus sealed tube	*R*_int_ = 0.054
Monochromator: graphite	θ_max_ = 25.2º
*T* = 295(2) K	θ_min_ = 3.0º
ω scans	*h* = −9→8
Absorption correction: none	*k* = −15→15
19548 measured reflections	*l* = −27→27
2559 independent reflections	

### Refinement

**Table:**

Refinement on *F*^2^	Secondary atom site location: difference Fourier map
Least-squares matrix: full	Hydrogen site location: inferred from neighbouring sites
*R*[*F*^2^ > 2σ(*F*^2^)] = 0.056	H-atom parameters constrained
*wR*(*F*^2^) = 0.151	*w* = 1/[σ^2^(*F*_o_^2^) + (0.0933*P*)^2^ + 0.2684*P*] where *P* = (*F*_o_^2^ + 2*F*_c_^2^)/3
*S* = 1.05	(Δ/σ)_max_ = 0.002
2559 reflections	Δρ_max_ = 0.30 e Å^−^^3^
293 parameters	Δρ_min_ = −0.18 e Å^−^^3^
6 restraints	Extinction correction: none
Primary atom site location: structure-invariant direct methods	

### Special details

**Table:**

Geometry. All e.s.d.'s (except the e.s.d. in the dihedral angle between two l.s. planes) are estimated using the full covariance matrix. The cell e.s.d.'s are taken into account individually in the estimation of e.s.d.'s in distances, angles and torsion angles; correlations between e.s.d.'s in cell parameters are only used when they are defined by crystal symmetry. An approximate (isotropic) treatment of cell e.s.d.'s is used for estimating e.s.d.'s involving l.s. planes.
Refinement. Refinement of *F*^2^ against ALL reflections. The weighted *R*-factor *wR* and goodness of fit *S* are based on *F*^2^, conventional *R*-factors *R* are based on *F*, with *F* set to zero for negative *F*^2^. The threshold expression of *F*^2^ > σ(*F*^2^) is used only for calculating *R*-factors(gt) *etc*. and is not relevant to the choice of reflections for refinement. *R*-factors based on *F*^2^ are statistically about twice as large as those based on *F*, and *R*- factors based on ALL data will be even larger.

### Fractional atomic coordinates and isotropic or equivalent isotropic displacement parameters (Å^2^)

**Table:**

	*x*	*y*	*z*	*U*_iso_*/*U*_eq_	Occ. (<1)
O1	−0.0903 (5)	0.5823 (4)	0.10016 (17)	0.1137 (15)	
O2	0.1457 (3)	0.82438 (19)	0.29175 (10)	0.0474 (6)	
O3	0.4884 (4)	0.8328 (2)	0.49749 (10)	0.0644 (8)	
O4	0.3946 (5)	0.8951 (3)	0.57892 (12)	0.0842 (10)	
O5	0.4856 (5)	0.9197 (3)	0.22555 (13)	0.0866 (11)	
O6	0.3136 (4)	0.8354 (2)	0.17081 (12)	0.0691 (8)	
C1	−0.0244 (6)	0.5948 (4)	0.1464 (2)	0.0742 (13)	
C2	0.1457 (6)	0.6130 (3)	0.15092 (19)	0.0642 (11)	
H2	0.2062	0.6122	0.1174	0.077*	
C3	0.2222 (5)	0.6310 (3)	0.20064 (16)	0.0476 (9)	
C4	0.1357 (4)	0.6367 (3)	0.25779 (16)	0.0481 (9)	
C5	−0.0438 (4)	0.6600 (4)	0.2471 (2)	0.0653 (11)	
H5A	−0.1024	0.6499	0.2826	0.078*	
H5B	−0.0552	0.7326	0.2361	0.078*	
C6	−0.1187 (6)	0.5921 (4)	0.2008 (2)	0.0759 (13)	
H6A	−0.1249	0.5208	0.2145	0.091*	
H6B	−0.2272	0.6160	0.1933	0.091*	
C7	0.4024 (4)	0.6423 (3)	0.20023 (16)	0.0515 (9)	
H7A	0.4385	0.6512	0.1610	0.062*	
H7B	0.4502	0.5787	0.2149	0.062*	
C8	0.4618 (4)	0.7341 (3)	0.23596 (14)	0.0465 (9)	
H8	0.5790	0.7285	0.2383	0.056*	
C9	0.3961 (4)	0.7217 (3)	0.29729 (14)	0.0432 (8)	
H9	0.4299	0.6526	0.3105	0.052*	
C10	0.2131 (4)	0.7204 (3)	0.29597 (15)	0.0438 (8)	
C11	0.1258 (4)	0.7665 (3)	0.34520 (16)	0.0467 (9)	
H11	0.0178	0.7384	0.3516	0.056*	
C12	0.2055 (4)	0.8119 (3)	0.39783 (15)	0.0508 (9)	
H12A	0.1525	0.7852	0.4318	0.061*	
H12B	0.1925	0.8871	0.3975	0.061*	
C13	0.3852 (5)	0.7856 (3)	0.40082 (14)	0.0474 (9)	
C14	0.4600 (4)	0.7996 (3)	0.34071 (14)	0.0451 (9)	
H14	0.4309	0.8694	0.3271	0.054*	
C15	0.6431 (5)	0.7989 (4)	0.35129 (17)	0.0646 (12)	
H15A	0.6865	0.7293	0.3463	0.078*	
H15B	0.6972	0.8458	0.3250	0.078*	
C16	0.6647 (5)	0.8359 (5)	0.41383 (17)	0.0721 (13)	
H16A	0.7153	0.7820	0.4368	0.086*	
H16B	0.7307	0.8981	0.4153	0.086*	
C17	0.4943 (5)	0.8590 (3)	0.43599 (14)	0.0534 (10)	
C18	0.4499 (5)	0.9740 (3)	0.43479 (17)	0.0578 (10)	
H18A	0.3843	0.9900	0.4015	0.069*	
H18B	0.5455	1.0173	0.4339	0.069*	
C19	0.3553 (7)	0.9913 (4)	0.49035 (16)	0.0727 (13)	
H19A	0.3789	1.0590	0.5069	0.087*	
H19B	0.2406	0.9858	0.4837	0.087*	
C20	0.4135 (6)	0.9058 (3)	0.52799 (16)	0.0629 (11)	
C21	0.1522 (6)	0.5303 (3)	0.28953 (19)	0.0659 (11)	
H21A	0.2639	0.5144	0.2949	0.099*	
H21B	0.1027	0.4765	0.2671	0.099*	
H21C	0.1002	0.5346	0.3262	0.099*	
C22	0.4032 (6)	0.6722 (3)	0.42265 (17)	0.0648 (11)	
H22A	0.3698	0.6684	0.4620	0.097*	
H22B	0.5135	0.6511	0.4196	0.097*	
H22C	0.3374	0.6268	0.3999	0.097*	
C23	0.4237 (5)	0.8399 (3)	0.21110 (15)	0.0503 (9)	
C24	0.2627 (8)	0.9320 (4)	0.1450 (2)	0.0944 (18)	
H24A	0.2670	0.9867	0.1731	0.142*	
H24B	0.1546	0.9249	0.1312	0.142*	
H24C	0.3326	0.9489	0.1136	0.142*	
O8	0.547 (6)	0.823 (3)	0.0144 (17)	0.431 (13)*	0.50
H8A	0.5024	0.8527	−0.0201	0.646*	0.50
C81	0.715 (6)	0.815 (6)	0.021 (2)	0.431 (13)*	0.50
H81A	0.7682	0.8468	−0.0114	0.517*	0.50
H81B	0.7455	0.7423	0.0227	0.517*	0.50
C82	0.769 (7)	0.868 (5)	0.075 (2)	0.431 (13)*	0.50
H82A	0.8188	0.8181	0.1000	0.646*	0.50
H82B	0.6778	0.8983	0.0940	0.646*	0.50
H82C	0.8447	0.9219	0.0657	0.646*	0.50
O9	0.632 (5)	0.917 (4)	0.0182 (15)	0.431 (13)*	0.50
H9A	0.5650	0.9195	−0.0163	0.646*	0.50
C91	0.625 (7)	0.824 (4)	0.050 (3)	0.431 (13)*	0.50
H91A	0.6519	0.7660	0.0249	0.517*	0.50
H91B	0.5155	0.8136	0.0632	0.517*	0.50
C92	0.735 (7)	0.825 (5)	0.100 (2)	0.431 (13)*	0.50
H92A	0.7614	0.7541	0.1100	0.646*	0.50
H92B	0.6839	0.8584	0.1315	0.646*	0.50
H92C	0.8316	0.8614	0.0898	0.646*	0.50

### Atomic displacement parameters (Å^2^)

**Table:**

	*U*^11^	*U*^22^	*U*^33^	*U*^12^	*U*^13^	*U*^23^
O1	0.100 (3)	0.143 (4)	0.098 (3)	−0.039 (3)	−0.033 (2)	−0.015 (3)
O2	0.0473 (14)	0.0399 (13)	0.0551 (13)	0.0060 (11)	0.0018 (12)	0.0004 (11)
O3	0.091 (2)	0.0635 (17)	0.0388 (13)	0.0233 (17)	−0.0092 (14)	−0.0007 (13)
O4	0.127 (3)	0.079 (2)	0.0468 (16)	0.021 (2)	0.0019 (18)	0.0003 (15)
O5	0.114 (3)	0.072 (2)	0.0730 (19)	−0.039 (2)	−0.005 (2)	0.0047 (17)
O6	0.087 (2)	0.0521 (17)	0.0681 (16)	−0.0022 (17)	−0.0233 (16)	0.0092 (14)
C1	0.070 (3)	0.068 (3)	0.084 (3)	−0.016 (2)	−0.015 (3)	−0.018 (3)
C2	0.070 (3)	0.062 (3)	0.061 (2)	−0.006 (2)	−0.003 (2)	−0.011 (2)
C3	0.053 (2)	0.0314 (18)	0.058 (2)	0.0005 (16)	0.0028 (19)	−0.0040 (16)
C4	0.0421 (18)	0.041 (2)	0.062 (2)	0.0008 (16)	0.0030 (18)	−0.0031 (16)
C5	0.045 (2)	0.060 (3)	0.091 (3)	−0.001 (2)	−0.001 (2)	−0.023 (2)
C6	0.053 (2)	0.063 (3)	0.112 (4)	−0.009 (2)	−0.008 (3)	−0.023 (3)
C7	0.052 (2)	0.054 (2)	0.0484 (19)	0.0091 (18)	0.0050 (18)	−0.0047 (18)
C8	0.0345 (18)	0.061 (2)	0.0443 (19)	0.0012 (17)	0.0017 (16)	−0.0036 (17)
C9	0.0380 (17)	0.047 (2)	0.0443 (17)	0.0067 (15)	0.0050 (16)	0.0000 (16)
C10	0.0453 (18)	0.0397 (19)	0.0464 (18)	0.0024 (16)	0.0052 (17)	0.0026 (16)
C11	0.0408 (19)	0.042 (2)	0.057 (2)	0.0010 (16)	0.0100 (17)	0.0014 (17)
C12	0.054 (2)	0.052 (2)	0.0461 (18)	0.0068 (19)	0.0109 (18)	−0.0004 (17)
C13	0.052 (2)	0.052 (2)	0.0380 (17)	0.0077 (17)	0.0021 (17)	−0.0009 (16)
C14	0.0380 (18)	0.058 (2)	0.0397 (17)	0.0012 (17)	0.0022 (15)	0.0004 (16)
C15	0.045 (2)	0.095 (4)	0.054 (2)	0.007 (2)	−0.0038 (19)	−0.012 (2)
C16	0.053 (2)	0.103 (4)	0.060 (2)	0.017 (3)	−0.010 (2)	−0.016 (3)
C17	0.063 (2)	0.060 (2)	0.0365 (17)	0.008 (2)	−0.0021 (17)	0.0003 (17)
C18	0.066 (3)	0.056 (2)	0.051 (2)	−0.001 (2)	−0.0043 (19)	0.0061 (19)
C19	0.105 (4)	0.061 (3)	0.052 (2)	0.016 (3)	−0.004 (2)	0.000 (2)
C20	0.086 (3)	0.059 (3)	0.043 (2)	0.010 (2)	−0.005 (2)	−0.0002 (19)
C21	0.071 (3)	0.048 (2)	0.079 (3)	−0.001 (2)	0.014 (2)	0.005 (2)
C22	0.088 (3)	0.056 (2)	0.051 (2)	0.017 (2)	0.004 (2)	0.0080 (19)
C23	0.053 (2)	0.055 (2)	0.0424 (18)	−0.0129 (19)	0.0082 (17)	−0.0015 (17)
C24	0.122 (5)	0.071 (3)	0.090 (3)	0.020 (3)	−0.018 (3)	0.018 (3)

### Geometric parameters (Å, °)

**Table:**

O1—C1	1.221 (6)	C14—C15	1.545 (5)
O2—C10	1.451 (4)	C14—H14	0.9800
O2—C11	1.460 (4)	C15—C16	1.544 (6)
O3—C20	1.331 (5)	C15—H15A	0.9700
O3—C17	1.474 (4)	C15—H15B	0.9700
O4—C20	1.206 (5)	C16—C17	1.538 (6)
O5—C23	1.195 (5)	C16—H16A	0.9700
O6—C23	1.314 (5)	C16—H16B	0.9700
O6—C24	1.441 (5)	C17—C18	1.522 (6)
C1—C2	1.438 (7)	C18—C19	1.532 (6)
C1—C6	1.492 (7)	C18—H18A	0.9700
C2—C3	1.343 (6)	C18—H18B	0.9700
C2—H2	0.9300	C19—C20	1.486 (6)
C3—C7	1.508 (5)	C19—H19A	0.9700
C3—C4	1.516 (5)	C19—H19B	0.9700
C4—C10	1.536 (5)	C21—H21A	0.9600
C4—C5	1.544 (5)	C21—H21B	0.9600
C4—C21	1.559 (5)	C21—H21C	0.9600
C5—C6	1.520 (6)	C22—H22A	0.9600
C5—H5A	0.9700	C22—H22B	0.9600
C5—H5B	0.9700	C22—H22C	0.9600
C6—H6A	0.9700	C24—H24A	0.9600
C6—H6B	0.9700	C24—H24B	0.9600
C7—C8	1.525 (5)	C24—H24C	0.9600
C7—H7A	0.9700	O8—C81	1.409 (11)
C7—H7B	0.9700	O8—H8A	0.9668
C8—C23	1.510 (5)	C81—C82	1.494 (11)
C8—C9	1.539 (5)	C81—H81A	0.9700
C8—H8	0.9800	C81—H81B	0.9700
C9—C14	1.519 (5)	C82—H82A	0.9600
C9—C10	1.523 (5)	C82—H82B	0.9600
C9—H9	0.9800	C82—H82C	0.9600
C10—C11	1.482 (5)	O9—C91	1.403 (11)
C11—C12	1.512 (5)	O9—H9A	0.9804
C11—H11	0.9800	C91—C92	1.483 (11)
C12—C13	1.535 (5)	C91—H91A	0.9700
C12—H12A	0.9700	C91—H91B	0.9700
C12—H12B	0.9700	C92—H92A	0.9600
C13—C14	1.544 (5)	C92—H92B	0.9600
C13—C17	1.545 (6)	C92—H92C	0.9600
C13—C22	1.548 (6)		
			
C10—O2—C11	61.2 (2)	C14—C15—H15A	110.7
C20—O3—C17	112.0 (3)	C16—C15—H15B	110.7
C23—O6—C24	117.8 (4)	C14—C15—H15B	110.7
O1—C1—C2	121.9 (5)	H15A—C15—H15B	108.8
O1—C1—C6	120.8 (4)	C17—C16—C15	105.6 (3)
C2—C1—C6	117.3 (4)	C17—C16—H16A	110.6
C3—C2—C1	123.9 (4)	C15—C16—H16A	110.6
C3—C2—H2	118.1	C17—C16—H16B	110.6
C1—C2—H2	118.1	C15—C16—H16B	110.6
C2—C3—C7	118.9 (4)	H16A—C16—H16B	108.7
C2—C3—C4	122.8 (3)	O3—C17—C18	103.4 (3)
C7—C3—C4	118.3 (3)	O3—C17—C16	108.3 (3)
C3—C4—C10	110.1 (3)	C18—C17—C16	113.9 (4)
C3—C4—C5	109.1 (3)	O3—C17—C13	111.0 (3)
C10—C4—C5	111.4 (3)	C18—C17—C13	116.0 (3)
C3—C4—C21	109.4 (3)	C16—C17—C13	104.3 (3)
C10—C4—C21	107.4 (3)	C17—C18—C19	104.4 (3)
C5—C4—C21	109.4 (3)	C17—C18—H18A	110.9
C6—C5—C4	113.6 (3)	C19—C18—H18A	110.9
C6—C5—H5A	108.8	C17—C18—H18B	110.9
C4—C5—H5A	108.8	C19—C18—H18B	110.9
C6—C5—H5B	108.8	H18A—C18—H18B	108.9
C4—C5—H5B	108.8	C20—C19—C18	103.0 (4)
H5A—C5—H5B	107.7	C20—C19—H19A	111.2
C1—C6—C5	112.0 (4)	C18—C19—H19A	111.2
C1—C6—H6A	109.2	C20—C19—H19B	111.2
C5—C6—H6A	109.2	C18—C19—H19B	111.2
C1—C6—H6B	109.2	H19A—C19—H19B	109.1
C5—C6—H6B	109.2	O4—C20—O3	120.5 (4)
H6A—C6—H6B	107.9	O4—C20—C19	128.5 (4)
C3—C7—C8	113.1 (3)	O3—C20—C19	110.9 (3)
C3—C7—H7A	109.0	C4—C21—H21A	109.5
C8—C7—H7A	109.0	C4—C21—H21B	109.5
C3—C7—H7B	109.0	H21A—C21—H21B	109.5
C8—C7—H7B	109.0	C4—C21—H21C	109.5
H7A—C7—H7B	107.8	H21A—C21—H21C	109.5
C23—C8—C7	114.6 (3)	H21B—C21—H21C	109.5
C23—C8—C9	112.0 (3)	C13—C22—H22A	109.5
C7—C8—C9	108.2 (3)	C13—C22—H22B	109.5
C23—C8—H8	107.2	H22A—C22—H22B	109.5
C7—C8—H8	107.2	C13—C22—H22C	109.5
C9—C8—H8	107.2	H22A—C22—H22C	109.5
C14—C9—C10	111.8 (3)	H22B—C22—H22C	109.5
C14—C9—C8	115.3 (3)	O5—C23—O6	122.7 (4)
C10—C9—C8	109.7 (3)	O5—C23—C8	124.9 (4)
C14—C9—H9	106.5	O6—C23—C8	112.4 (3)
C10—C9—H9	106.5	O6—C24—H24A	109.5
C8—C9—H9	106.5	O6—C24—H24B	109.5
O2—C10—C11	59.7 (2)	H24A—C24—H24B	109.5
O2—C10—C9	112.2 (3)	O6—C24—H24C	109.5
C11—C10—C9	118.1 (3)	H24A—C24—H24C	109.5
O2—C10—C4	116.2 (3)	H24B—C24—H24C	109.5
C11—C10—C4	121.5 (3)	C81—O8—H8A	120.4
C9—C10—C4	116.0 (3)	C81—O8—H9A	91.0
O2—C11—C10	59.1 (2)	O8—C81—C82	111.1 (11)
O2—C11—C12	116.5 (3)	O8—C81—H81A	109.4
C10—C11—C12	124.6 (3)	C82—C81—H81A	109.4
O2—C11—H11	114.9	O8—C81—H81B	109.4
C10—C11—H11	114.9	C82—C81—H81B	109.4
C12—C11—H11	114.9	H81A—C81—H81B	108.0
C11—C12—C13	112.3 (3)	C81—C82—H82A	109.5
C11—C12—H12A	109.1	C81—C82—H82B	109.5
C13—C12—H12A	109.1	H82A—C82—H82B	109.5
C11—C12—H12B	109.1	C81—C82—H82C	109.5
C13—C12—H12B	109.1	H82A—C82—H82C	109.5
H12A—C12—H12B	107.9	H82B—C82—H82C	109.5
C12—C13—C14	109.0 (3)	C91—O9—H9A	115.6
C12—C13—C17	117.6 (3)	O9—C91—C92	112.3 (12)
C14—C13—C17	100.0 (3)	O9—C91—H91A	109.1
C12—C13—C22	108.5 (3)	C92—C91—H91A	109.1
C14—C13—C22	111.7 (3)	O9—C91—H91B	109.1
C17—C13—C22	110.0 (3)	C92—C91—H91B	109.1
C9—C14—C13	112.8 (3)	H91A—C91—H91B	107.9
C9—C14—C15	116.6 (3)	C91—C92—H92A	109.5
C13—C14—C15	104.6 (3)	C91—C92—H92B	109.5
C9—C14—H14	107.5	H92A—C92—H92B	109.5
C13—C14—H14	107.5	C91—C92—H92C	109.5
C15—C14—H14	107.5	H92A—C92—H92C	109.5
C16—C15—C14	105.3 (3)	H92B—C92—H92C	109.5
C16—C15—H15A	110.7		
			
O1—C1—C2—C3	177.4 (5)	O2—C11—C12—C13	−81.0 (4)
C6—C1—C2—C3	−2.3 (7)	C10—C11—C12—C13	−11.7 (5)
C1—C2—C3—C7	176.2 (4)	C11—C12—C13—C14	43.1 (4)
C1—C2—C3—C4	−1.5 (7)	C11—C12—C13—C17	155.9 (3)
C2—C3—C4—C10	−143.4 (4)	C11—C12—C13—C22	−78.6 (4)
C7—C3—C4—C10	38.9 (4)	C10—C9—C14—C13	51.8 (4)
C2—C3—C4—C5	−20.9 (5)	C8—C9—C14—C13	178.0 (3)
C7—C3—C4—C5	161.4 (3)	C10—C9—C14—C15	172.9 (3)
C2—C3—C4—C21	98.7 (4)	C8—C9—C14—C15	−60.9 (5)
C7—C3—C4—C21	−79.0 (4)	C12—C13—C14—C9	−66.2 (4)
C3—C4—C5—C6	47.3 (5)	C17—C13—C14—C9	169.9 (3)
C10—C4—C5—C6	169.0 (4)	C22—C13—C14—C9	53.7 (4)
C21—C4—C5—C6	−72.4 (5)	C12—C13—C14—C15	166.1 (3)
O1—C1—C6—C5	−150.7 (5)	C17—C13—C14—C15	42.2 (4)
C2—C1—C6—C5	29.0 (7)	C22—C13—C14—C15	−74.1 (4)
C4—C5—C6—C1	−52.5 (6)	C9—C14—C15—C16	−151.0 (4)
C2—C3—C7—C8	134.9 (4)	C13—C14—C15—C16	−25.7 (5)
C4—C3—C7—C8	−47.3 (5)	C14—C15—C16—C17	−1.4 (5)
C3—C7—C8—C23	−70.2 (4)	C20—O3—C17—C18	14.6 (5)
C3—C7—C8—C9	55.7 (4)	C20—O3—C17—C16	135.7 (4)
C23—C8—C9—C14	−59.7 (4)	C20—O3—C17—C13	−110.5 (4)
C7—C8—C9—C14	172.9 (3)	C15—C16—C17—O3	146.3 (4)
C23—C8—C9—C10	67.5 (4)	C15—C16—C17—C18	−99.4 (4)
C7—C8—C9—C10	−59.9 (4)	C15—C16—C17—C13	28.0 (5)
C11—O2—C10—C9	−110.5 (3)	C12—C13—C17—O3	82.8 (4)
C11—O2—C10—C4	112.7 (4)	C14—C13—C17—O3	−159.5 (3)
C14—C9—C10—O2	48.9 (4)	C22—C13—C17—O3	−41.9 (4)
C8—C9—C10—O2	−80.2 (4)	C12—C13—C17—C18	−34.7 (5)
C14—C9—C10—C11	−17.5 (5)	C14—C13—C17—C18	83.0 (4)
C8—C9—C10—C11	−146.7 (3)	C22—C13—C17—C18	−159.5 (3)
C14—C9—C10—C4	−174.2 (3)	C12—C13—C17—C16	−160.8 (3)
C8—C9—C10—C4	56.6 (4)	C14—C13—C17—C16	−43.1 (4)
C3—C4—C10—O2	91.2 (4)	C22—C13—C17—C16	74.5 (4)
C5—C4—C10—O2	−30.0 (5)	O3—C17—C18—C19	−23.4 (4)
C21—C4—C10—O2	−149.7 (3)	C16—C17—C18—C19	−140.6 (4)
C3—C4—C10—C11	160.2 (3)	C13—C17—C18—C19	98.3 (4)
C5—C4—C10—C11	39.1 (5)	C17—C18—C19—C20	23.9 (5)
C21—C4—C10—C11	−80.7 (4)	C17—O3—C20—O4	178.1 (4)
C3—C4—C10—C9	−44.0 (4)	C17—O3—C20—C19	1.0 (5)
C5—C4—C10—C9	−165.1 (3)	C18—C19—C20—O4	167.2 (5)
C21—C4—C10—C9	75.1 (4)	C18—C19—C20—O3	−16.1 (5)
C10—O2—C11—C12	116.1 (3)	C24—O6—C23—O5	−2.4 (6)
C9—C10—C11—O2	100.7 (3)	C24—O6—C23—C8	177.9 (4)
C4—C10—C11—O2	−104.0 (3)	C7—C8—C23—O5	−164.2 (4)
O2—C10—C11—C12	−102.7 (4)	C9—C8—C23—O5	72.0 (5)
C9—C10—C11—C12	−2.0 (5)	C7—C8—C23—O6	15.5 (4)
C4—C10—C11—C12	153.4 (3)	C9—C8—C23—O6	−108.3 (3)

### Hydrogen-bond geometry (Å, °)

**Table:**

*D*—H···*A*	*D*—H	H···*A*	*D*···*A*	*D*—H···*A*
O8—H8A···O1^i^	0.96	2.19	3.15 (4)	178
O9—H9A···O1^i^	0.98	2.34	3.32 (4)	177
C6—H6A···O2^ii^	0.97	2.53	3.447 (6)	157
C7—H7B···O5^iii^	0.97	2.52	3.467 (5)	164
C11—H11···O4^iv^	0.98	2.57	3.337 (5)	135
C14—H14···O5	0.98	2.50	3.103 (5)	120
C21—H21A···O5^iii^	0.96	2.46	3.351 (6)	154
C92—H92A···O1^v^	0.96	2.54	3.44 (6)	155

Symmetry codes: (i) *x*+1/2, −*y*+3/2, −*z*; (ii) −*x*, *y*−1/2, −*z*+1/2; (iii) −*x*+1, *y*−1/2, −*z*+1/2; (iv) *x*−1/2, −*y*+3/2, −*z*+1; (v) *x*+1, *y*, *z*.
